# Mid-trimester cesarean scar pregnancy: a case report

**DOI:** 10.1186/s40738-021-00103-9

**Published:** 2021-04-23

**Authors:** Mary Louise Fowler, Sarah Little, Michael Muto, Shruthi Mahalingaiah

**Affiliations:** 1 Department of Obstetrics and Gynecology, Boston Medical Center, 85 East Concord Street, 6th Floor, Boston, MA 02118 USA; 2 Department of Maternal Fetal Medicine, Brigham and Women’s Hospital, 75 Francis Street, Boston, MA 02115 USA; 3 Division of Gynecologic Oncology, Brigham and Women’s Hospital, 75 Francis Street, Boston, MA 02115 USA; 4 Division of Reproductive Endocrinology and Infertility (REI), Massachusetts General Hospital, Yawkey Center – 10th Floor, Boston, MA 02114 USA; 5 Department of Environmental Health, Harvard TH Chan School of Public Health, 677 Huntington Avenue, Building 1, 14th Floor, Boston, MA 02115 USA

**Keywords:** Cesarean scar pregnancy, Cesarean ectopic, Hysterectomy

## Abstract

**Background:**

This article reports a unique case of cesarean scar pregnancy, demonstrating importance of early management and diagnosis.

**Case presentation:**

A 30-year-old pregnant woman with prior history of two cesarean sections found to have cesarean scar pregnancy at approximately 13 weeks’ gestation and underwent a gravid hysterectomy.

**Conclusions:**

While rare, cesarean scar pregnancies should be considered on the differential diagnosis of any pregnant patient with history of cesarean section who presents in early pregnancy with vaginal bleeding and/or cramping. Given the increased rates of cesarean sections in the times of COVID-19, one may anticipate seeing more cases of cesarean scar pregnancies.

## Background

Cesarean scar pregnancy (CSP), while rare, with a frequency of approximately 1:800 to 1:2500 of all pregnancies [1], has an increased prevalence over the last two decades, secondary to an increase in primary and repeat cesarean sections. Additionally, in the times of the COVID pandemic, there has been noted to be an increase in cesarean deliveries, with one study noting that 93% of COVID-positive pregnant patients underwent a cesarean section, and 61% of these had the procedure performed due to concern about effects of COVID-19 on the pregnancy [2]. These pregnancies can result in significant morbidity and mortality, including uterine rupture and may require emergent hysterectomy if not recognized early. Early diagnosis of a CSP is crucial, however may often be missed or misdiagnosed as either a cervical pregnancy or an incomplete abortion. While diagnosis has improved with the technological improvement in ultrasonography, optimal management of CSP is unknown and a standard of care has not been identified. We present a case of CSP diagnosed in the second trimester and subsequent management.

## Case presentation

**Fig. 1:**
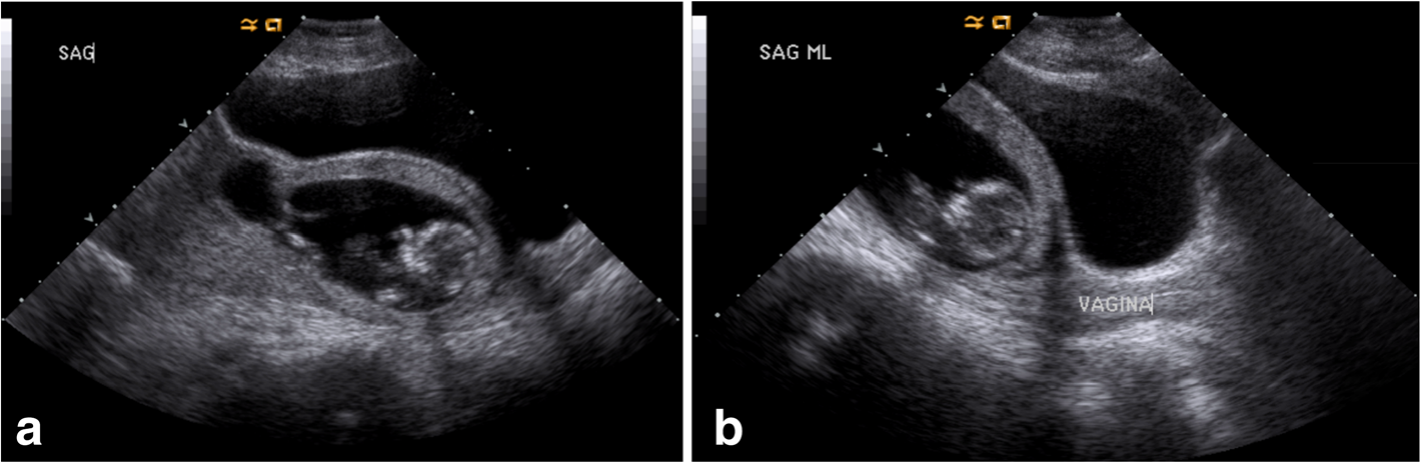
Sagittal transvaginal ultrasounds demonstrating cesarean scar ectopic pregnancy located in close proximity to bladder (anteriorly). Image on the left shows a very thin myometrium (2-5 mm) between gestational sac and bladder

This is a case report of a 30-year-old gravida 4 para 2 aborta 1 who initially presented to an outside hospital with complaint of a rash and was incidentally found to have a positive beta-human chorionic gonadotropin (beta-hCG). In further discussion with the patient, she reported a history of some crampy abdominal pain. Her obstetric history is significant for 1 prior miscarriage as well as 2 cesarean sections, indications were breech presentation and a subsequent planned repeat. She had no past medical history and no additional past surgical history. A first trimester ultrasound at an outside hospital revealed a low-lying embryo, after which she was transferred to our hospital for repeat ultrasound, shown in Fig. 1, and consultation on further management. Given temporality of this case from timing of this write up consent from the patient was not able to be obtained.

**Fig. 2:**
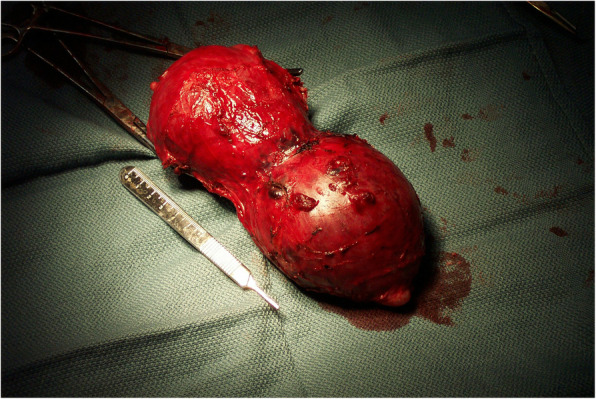
Gross pathology demonstrating cesarean scar ectopic pregnancy

On presentation, physical exam was notable for a soft, non-tender gravid abdomen with a well-healed Pfannenstiel scar. Vital signs were within normal limits, with a heart rate of 92 bpm and blood pressure of 116/66 mm HG. Repeat ultrasound demonstrated a gestational sac with an embryo measuring 64 mm, corresponding to 13 weeks gestational age, that was implanted in the lower uterine segment likely within the cesarean section scar. There was no myometrium seen surrounding the sac. A magnetic resonance imaging (MRI) to further evaluate these findings was attempted, but the patient sustained an anxiety attack in the scanner and the exam was unable to be completed. She was admitted to the hospital with the diagnosis of cesarean scar pregnancy for inpatient observation to determine optimal management. Laboratory evaluation revealed a preoperative hematocrit of 41%, which downtrended to 34%. She was counseled regarding her options and given a pre-operative social work consult. Ultimately, she underwent a gravid hysterectomy. Estimated blood loss from the procedure was 50 cc and her post-operative course was unremarkable. She was discharged on post-operative day 3 without complication. Gross pathology demonstrating the cesarean scar ectopic pregnancy is shown in Fig. 2.

## Discussion and conclusions

Cesarean scar pregnancies (CSP) are a unique form of a pregnancy – they are not truly ectopic as they exist within the uterine corpus yet have potentially dangerous outcomes for women, similar to those of ectopics, if not discovered early. These complications include uterine rupture causing significant hemorrhage, hemorrhagic shock, blood transfusion, need for possible hysterectomy, loss of pregnancy, and death. In addition to this, CSP are unique for each patient and exist as a continuum – ranging from partial implantation over the prior cesarean scar to fully embedded and extending through to the parametrium [3]. For women who have had a cesarean section, the frequency of CSP is approximately 0.15%, which accounts for 6.1% of all ectopic pregnancies in women with at least one cesarean section. While history of a cesarean section is a risk factor, the number of prior cesarean sections does not necessarily correlate with risk for CSP. Additionally, CSP has been reported after other types of uterine surgery, such as myomectomy, as well as previously abnormally placentation, uterine instrumentation, and in-vitro fertilization [4]. There is some data that women who had an elective cesarean section for breech presentation are at higher risk due to poor formation of the lower uterine segment [5]. The mean gestational age is 7 weeks 5 days and most commonly the presenting sign was vaginal bleeding, often without abdominal pain. According to Zhang et al., early CSP can be misdiagnosed as normal intrauterine pregnancy, missed abortion, inevitable abortion, or cervical pregnancy [6]. As a result, significant delay between initial presentation and management of CSP can occur.

Diagnosis of CSP is made by visualization of a mass embedded within the hysterotomy scar with thinning of a visible defect in the myometrium between the gestational sac and bladder as well as a concomitant empty endometrial cavity [1]. Transvaginal and abdominal ultrasound is the most common imaging modality for diagnosis, but further detail may be obtained from MRI. A case series has suggested that 66% of CSPs have a thinning myometrium that is less than 5 mm in thickness [7]. Color Doppler evidence of a high-speed peritrophoblastic flow and low resistance near the hysterotomy scar may be important prognostic indicators for possible treatment complications [8].

Given rarity of CSP as well as variety in presentations, optimal management has not been well established. Through an in-depth review of 751 cases of cesarean scar pregnancies, Timor-Tritsch and Monteagudo identified 31 different primary treatment modalities for CSP described in the literature [9] including expectant management, methotrexate (either systemic or local), suction curettage, hysteroscopy, laparoscopy, and may require hysterectomy. Many of the more conservative treatment modalities are likely only effective when recognized early in gestational age. Additionally, suction curettage has a high risk of incomplete evacuation, bladder injury, or perforation through the previous cesarean section scar given thinned out myometrial tissue. Timor-Tritsch and Monteagudo highlighted data that shows a trend towards improved outcome for earlier diagnosis and treatment. For example, of the 20 cases with gestational ages greater than or equal to 10 weeks, 16 of them (80%) had complications, defined as immediate or delayed need for a secondary treatment.

For this patient, because she presented to care and was found to be have a CSP at 13 weeks, the options for management were much more limited than those with CSP at earlier gestations. In a study by Timor-Tritsch et al., they present a case series of 60 CSP at a tertiary care center with various modes of management [10]. The gestational ages ranged from 5w4d to 14w. Eleven of the 57 patients (19.2%) ultimately required a hysterectomy. Of these 11, 3 had hysterectomies for placenta percreta at the time of live delivery, 5 had second-trimester complications all leading to hysterectomy (including 3 uterine rupture, 1 uterine dehiscence, and 1 bulging membranes), 2 had unsuccessful uterine artery embolization of arteriovenous malformation followed by a hysterectomy and 1 requested for a hysterectomy after a late-developing arteriovenous malformation. A retrospective review by Ballas et al. [11] reported on 10 cases of women undergoing cesarean deliveries for morbidly adherent placentas that required hysterectomies. At gestational ages of 8 to 14 weeks, the pregnancies fulfilled the diagnostic criteria of cesarean scar pregnancies. Therefore, these women delivered live offspring with unavoidable peripartum hysterectomies, which have much higher risk of morbidity and mortality than planned hysterectomy under more controlled conditions such as this patient. One factor that limited alternative modes of treatment was the near lack of existence of myometrium between the gestational sac and bladder.

This case serves as an important reminder to keep cesarean scar pregnancies on the differential for any pregnant patient with prior history of a cesarean section who presents to the hospital with vaginal bleeding or has concerning signs on ultrasound of a low-lying embryo. Additionally, women should be counseled after a primary cesarean section about the rare but increased risk of a CSP due to prior uterine surgery and to seek evaluation with vaginal bleeding early in a subsequent pregnancy. Primary cesarean rates had already been on the rise for decades, but now more-so in the time of COVID, therefore one should look to diagnose this as early as possible to provide an opportunity for fertility-sparing treatment.

## Data Availability

Not Applicable.

## Abbreviations

CSP: Cesarean scar pregnancy
Beta-hCG: Beta-human chorionic gonadotropin
MRI: Magnetic resonance imaging
